# Consensus Statement on the Management of Waldenström Macroglobulinemia Patients During the COVID-19 Pandemic

**DOI:** 10.1097/HS9.0000000000000433

**Published:** 2020-07-30

**Authors:** Dipti Talaulikar, Ranjana H. Advani, Andrew R. Branagan, Christian Buske, Meletios A. Dimopoulos, Shirley D'Sa, Maria J. Kersten, Veronique Leblond, Monique C. Minnema, Roger G. Owen, Maria Lia Palomba, Alessandra Tedeschi, Judith Trotman, Marzia Varettoni, Josephine M. Vos, Steven P. Treon, Efstathios Kastritis, Jorge J. Castillo

**Affiliations:** 1 Department of Hematology, Canberra Hospital and College of Health and Medicine, Australian National University, Canberra, Australia; 2 Department of Medicine, Stanford University Medical Center, Stanford, California, USA; 3 Division of Hematology and Oncology, Massachusetts General Hospital, Harvard Medical School, Boston, Massachusetts, USA; 4 Comprehensive Cancer Center Ulm, Institute of Experimental Cancer Research, University Hospital Ulm, Ulm, Germany; 5 Department of Clinical Therapeutics, National and Kapodistrian University of Athens, Athens, Greece; 6 Waldenström Clinic, Cancer Division, University College London Hospitals NHS Foundation Trust, London, UK; 7 Department of Hematology, Cancer Center Amsterdam and LYMMCARE (Lymphoma and Myeloma Center Amsterdam), University of Amsterdam, Amsterdam, The Netherlands; 8 Service of Clinical Hematology, Pitié-Salpêtrière Hospital, APHP, Sorbonne University, Paris, France; 9 Department of Hematology, University Medical Center Utrecht, University Utrecht, Utrecht, The Netherlands; 10 Haematological Malignancy Diagnostic Service, St James's University Hospital, Leeds, UK; 11 Lymphoma Service, Memorial Sloan Kettering Cancer Center, Weill Cornell Medical College, New York, New York, USA; 12 Department of Hematology, Niguarda Cancer Center, ASST Grande Ospedale Metropolitano Niguarda, Milan, Italy; 13 Department of Haematology, Concord Hospital, Department of Medicine, University of Sydney, Sydney, Australia; 14 Fondazione IRCCS Policlinico San Matteo, Pavia, Italy; 15 Department of Hematology and LYMMCARE (Lymphoma and Myeloma Center Amsterdam), Amsterdam UMC, location AMC, Amsterdam, The Netherlands; 16 Bing Center for Waldenström Macroglobulinemia, Dana-Farber Cancer Institute, Harvard Medical School, Boston, Massachusetts, USA.

## Abstract

In the light of the COVID-19 pandemic, the International Workshop on Waldenström Macroglobulinemia (IWWM) Treatment Recommendations Panel felt the need to provide a consensus statement for the management of Waldenström Macroglobulinemia (WM) patients during this challenging time. We followed the current recommendations by the American Society of Hematology, which have been modified accordingly to fit the specific realities associated with the management of WM. In this Consensus Statement, the Panel addresses questions related to treatment initiation, preferred therapies, minimizing visit to clinics and infusions centers, supportive care and guidance for WM patients in clinical trials. Finally, we also provide information on timing and appropriateness of testing and management of COVID-19 infected patients, as well as ways to get physicians and patients involved in registry studies that could help others.

## Introduction

The SARS-CoV-2-associated coronavirus disease 2019 (COVID-19) pandemic has affected patients with blood cancers across the globe. The American Society of Hematology has provided guidance on the management of various blood cancers and disorders during the time of this unique pandemic, in the form of questions and answers. There are mounting data suggesting an increased risk of COVID-19 infection in adults with cancer, and that patients with cancer have an increased risk of severe complications after contracting COVID-19,^1^ but it not clear if this is related to the increased age of cancer patients.^2,3^ Given that Waldenström Macroglobulinaemia (WM) is an indolent lymphoma with distinct features and treatment options,^4^ the International Workshop on Waldenström Macroglobulinemia provides the following consensus statement along a similar format. In particular, the immunomodulatory and anti-inflammatory responses of Bruton tyrosine kinase inhibitors (BTKi), and the potential risks of cytokine storm and hyperviscosity caused by BTKi withdrawal in worsening late complications of COVID-19 are highlighted. These international consensus recommendations are made based on current understanding of WM and of COVID-19 infection and must be interpreted and applied in the context of new data, as they become available.

**Q1: Should we be changing indications for therapy in Waldenström Macroglobulinemia (WM)?**

- The median age of presentation of WM is in the sixth and seventh decade of life, and patients are therefore at higher risk of developing severe complications from COVID-19 infections.^1^
- The threshold for initiating treatment should be high in this situation, and watchful waiting should be the preferred strategy whenever possible.
- Treatment is recommended in symptomatic patients, but if the indication for therapy is borderline, (eg, if the patient has mild, tolerable symptoms) treatment deferral and close monitoring may be prudent.
- For patients presenting with anemia and low iron levels, a trial of intravenous or oral iron (to reduce visits to healthcare centers) is recommended. Oral iron therapy is appropriate if there is an absolute iron deficiency with low ferritin, whereas function iron deficiency, which often manifests with normal ferritin, should preferably be managed with intravenous iron.
- Erythropoietin analogues should be considered where feasible when the only presentation is symptomatic anemia.

**Q2: Should we change our approach to initial therapy?**

- When first line treatment is indicated, depending on the local situation, chemoimmunotherapy and use of Bruton tyrosine kinase inhibitors (BTK) inhibitors^4^ remain reasonable options—for patients that cannot travel because of the risk of contracting COVID-19, oral therapy may be a better option.
- Many experts have concerns about the immunosuppressive properties of bendamustine^5^ and are recommending better tolerated regimens such as dexamethasone, rituximab and cyclophosphamide^6^ over bendamustine and rituximab.^7^
- The use of proteasome inhibitors (ie, bortezomib and carfilzomib) in combination with steroids and rituximab should be minimized, as it typically implies more frequent (weekly) visits to infusions centers, in addition to the immunosuppression associated with these agents.^8–10^
- Use of rituximab maintenance is to be avoided given the lack of survival benefit,^4^ the added burden of travelling to healthcare centers and the risk of associated immunosuppression.

**Q3: Should we change therapies for non-COVID-19 positive patients who have already started treatment?**

- The risk of COVID-19 infection and of morbidity and mortality in WM for various treatment regimens is unknown. There are currently no concrete data that suggests that cancer therapies should be ceased in patients on active treatment. However, treatment decisions require consideration based on patient's clinical status, degree of response, and risk of developing COVID-19 infection.
- Patients on BTK inhibitors should be continued on the possibility that these agents may reduce rates of COVID-19 pulmonary manifestations.^11,12^ Cessation of treatment also has a high risk of causing IgM rebound, potentially increasing risk of constitutional symptoms (which can be confused as COVID-19 related) and symptomatic hyperviscosity.^13^
- For patients who have already achieved an excellent response to R-chemotherapy, a reduced number of cycles may be considered. Alternatively, if feasible, a switch in therapy to less immunosuppressive or myelosuppressive approaches should be considered.

**Q4: Should we change therapy to minimize visits? For example, changing to oral or less frequent regimens?**

- Some experts are switching patients to oral options (eg, BTK inhibitors, rather than continuing intravenous chemotherapy) to reduce the risk of infection and limit the number of visits to the outpatient clinic.
- Omitting or delaying 1 or 2 cycles of immunotherapy, especially if a good response has already been achieved may be considered, especially in good/intermediate risk patients.
- Some patients may be eligible to receive up to a 3-month supply of their oral medication; this approach, with labs obtained locally and telehealth visits may allow patients to self-isolate at home.^14^
- Patients who are on “watchful waiting” may have visits delayed with telemedicine alternatives, with lab work obtained locally or delayed if risk is low. Home collection of blood samples should be used, if this service is provided by laboratories with appropriate social distancing measures.

**Q5: Should we change our approach to supportive care?**

- When choice of therapy is available, options that minimize outpatient visits are preferred. When feasible, select patients should be reviewed by telehealth to avoid clinic visits.^14^
- Growth factor support is recommended for select patients who are receiving bendamustine because of the increased risk of neutropenia.^5^
- Patients with comorbidities, recent infections, and low serum IgG levels, including those who have received rituximab, may benefit from immunoglobulin supplementation, if available. Subcutaneous administration or home treatment should be prioritized, if feasible.
- Consideration of a more liberal approach to antibiotic prophylactic regimens in consultation with Infectious Disease experts is recommended.
- Where indicated, routine vaccination against influenza and *Pneumococcus* should be continued despite reports of impaired responses.^15^

**Q6. What are the consensus treatment recommendations for relapsed/refractory disease?**

- Management of relapsed/refractory WM should be based on symptoms and indications for treatment as previously untreated patients. When possible, experts recommend delaying treatment as in treatment-naïve patient.
- The consensus treatment recommendations for this group mirror ones for treatment naïve WM.
- High dose chemotherapy followed by autologous stem cell infusion should be deferred.^14,16^

**Q7: What about patients enrolled in clinical trials?**

- Trial monitors and/or sponsors should be contacted to seek advice on management of patients who are on clinical trials.
- Supply of medication for longer durations to reduce patients having to come into healthcare centers should be considered.
- Local institutional review boards should be kept informed of any amendments or adverse events in trial patients.

**Q8: Is serological testing for COVID-19 likely to be affected by clonal IgM in patients with WM?**

- Serological lab tests for COVID-19 analyze COVID-19 specific IgM and will not be affected by the total IgM or paraprotein levels.
- Patients on rituximab or with hypogammaglobulinemia have been reported to have a blunted humoral response to vaccination,^17,18^ and the Panel recognized that there is a possibility of a false negative serological test after exposure to the COVID-19 virus in WM patients.

**Q9: Should I screen all WM patients for COVID-19?**

- The criteria and methods used to screen patients for COVID-19 vary from region to region and should be based on local government recommendations and guidelines.
- Similarly, prevention of COVID-19, the need and the way to self-isolate should be based on local government and health guidelines. The panel cautions against over interpretation about self-isolation in particular, especially for asymptomatic patients or untreated WM patients.

**Q10: What should I do if my patient presents with symptoms suggestive of COVID-19?**

- Testing and management of COVID-19 should be as per existing protocols within each country.
- Treatment of underlying WM will depend on clinical condition of patient and type of treatment (eg, continuation of BTK inhibitors treatment is recommended at this stage in patients who are mildly symptomatic, or asymptomatic).
- For more symptomatic patients, the dilemma is between a traditional approach to cease anti-cancer treatment, and of continuing treatment. This is especially pronounced for BTK inhibitors because of the potential immunosuppressive effect reported with ibrutinib.^15,19^ This may be of potential danger in mounting a response to COVID-19 but may be of benefit in reducing late and severe immune-mediated complications. There are preliminary data on the anti-inflammatory effects of Ibrutinib in murine lung,^12^ effect on suppression of inflammatory cytokines such as TNF, IL2RA, and CXCL13,^20^ and down regulatory effects on T cells and macrophages.^21^ A recent paper has shown that use of Ibrutinib in a small number of WM patients is associated with a low rate of COVID-19 related pulmonary complications.^11^ There is the additional risk of developing IgM flare and potentially worsening the clinical situation because of a cytokine storm if BTK inhibitors are withheld.^22^
- Close follow up, multidisciplinary approaches to management and agility in decision making is required.

**Q11: What else we can do to help?**

- Ongoing data collection and observations made during this pandemic may be of immense help in the future. If feasible, setting in processes to allow data collection during or after the acute period of the pandemic is crucial.
- The International Waldenström Macroglobulinemia Foundation has a wide network across the globe. WM patients and advocates play an active role in various aspects of disease management. Their help should be harnessed in dissemination of knowledge and in advocating for patients.

## Disclosures

DT received research funding and/or honoraria from Amgen, Janssen, Novartis, Roche and Takeda. RHA received research funding and/or honoraria from Genentech, Agensys, AstraZeneca, Autolus, Bayer, BMS, Celgene, Cell Medica, Forty Seven, Gilead, Infinity, Janssen, Kyowa Hakko Kirin, Kura, Merck, Millennium, Pharmacyclics, Regeneron, Roche, Seattle Genetics and Takeda. ARB received honoraria from Pharmacyclics. CB received research funding from Roche, Janssen, Bayer and MSD. MAD received honoraria from participation in advisory boards from Amgen, Takeda, Celgene, Janssen and BMS. SD received Honoraria and grant funding from Janssen, grant funding from BeiGene, Honoraria from Novartis, Advisory Board for Sanofi. EK received research funding and/or honoraria from Amgen, Genesis Pharma, Janssen, Takeda, Pfizer, Research Funding: Amgen, Jansen. MJK received research funding and/or honoraria from Novartis, Kite/Gilead, Celgene, Roche, BMS, Merck, Amgen, Janssen, Miltenyi Biotech. VL received honoraria from Abbvie, Beigene, Roche, Gilead, Janssen, Amgen and AstraZeneca. MCM received research funds and/or honoraria from Celgene/BMS, Janssen, Servier and Kite/Gilead. RGO received research funding from Aztra-Zeneca. MLP honoraria and/or research funding from Janssen and Pharmacyclics. JT received research funding from Pharmacyclics, Janssen, Roche, Celgene and Beigene. AT received honoraria from AbbVie, Janssen, Sunesis and AstraZeneca. SPT received honoraria and/or research funding from Pharmacyclics and BMS. MV received honoraria from Janssen and Roche.
